# Papillon-Lefèvre syndrome and squamous cell carcinoma: a case report

**DOI:** 10.4076/1757-1626-2-7067

**Published:** 2009-08-28

**Authors:** Sammy Al-Benna, Raphael Hasler, Ingo Stricker, Hans-Ulrich Steinau, Lars Steinstraesser

**Affiliations:** 1 Department of Plastic and Reconstructive Surgery, Department of Hand Surgery, Soft Tissue Tumour Reference Centre, BG University Hospital Bergmannsheil, Ruhr University BochumBuerkle-de-la Camp-Platz 1, 44789 BochumGermany; 2 Department of Pathology, Soft Tissue Tumour Reference Centre, BG University Hospital Bergmannsheil, Ruhr University BochumBuerkle-de-la-Camp-Platz 1, 44789 BochumGermany

## Abstract

Papillon-Lefèvre syndrome is a rare autosomal recessive genodermatosis characterised by palmoplantar hyperkeratosis and severe early-onset periodontitis. The development of malignant cutaneous neoplasms within the hyperkeratotic lesions of the syndrome is very rare. Here, we report on a 67-year-old German Caucasian male with Papillon-Lefèvre syndrome associated with recurrent squamous cell carcinoma. Treatment is symptomatic and not always satisfactory.

## Introduction

Papillon-Lefèvre syndrome (PLS) is a relatively rare autosomal recessive disorder. The 2 cardinal diagnostic features of the syndrome are palmoplantar keratosis and an early-onset form of aggressive periodontitis [1]. The palms and soles have a dry, red, and scaly appearance. Other areas, including cheeks, eyelids, labial commissures, legs, thighs, knees, and elbows, can be affected by the keratosis, although it varies significantly [1,2]. PLS usually manifests itself between the ages of 6 months to 4 years, coinciding with the eruption of primary teeth and leads to premature loss of both primary and permanent dentitions [1,3]. The soles of the feet are more severely affected than the hands, and erythema always precedes hyperkeratosis. The skin lesions are aggravated during cold weather and at times of severe periodontal involvement [1-3]. PLS is differentiated from other conditions showing similar oral or cutaneous clinical features by the presence of the palmoplantar hyperkeratosis [4]. Associated findings include hyperhidrosis, mild mental retardation, and frequent pyogenic infections of the skin and internal organs. Dural calcifications, especially of the tentorium and the falx cerebri have been noted in some cases [5]. Both sexes are equally affected. Consanguinity of the parents was observed in about one third of the cases described. Histopathologic findings are nonspecific, but show hyperkeratosis with irregular parakeratosis, acanthosis, and a moderate perivascular infiltrate. The skin changes rarely respond to classic external dermatotherapy. Retinoids improve the keratoderma and also reduce gingival inflammation, but usually the patient is totally edentulous by the late teen years despite rigorous periodontal therapy. In 1924, Papillon and Lefèvre first described this disease in a brother and sister [6]. Over 200 cases have been observed and the prevalence of affection is reported to be 1-4:1.000.000 with a carrier rate of 2 to 4 per 1000 [2,3,7] PLS is associated with cathepsin-C gene mutations and the gene responsible has been mapped to chromosome 11q14-q21, and more than 50 mutations in PLS patients have been identified in this gene; this may be due to specific virulent pathogens [8-10]. Variation in the clinical presentation of PLS has been observed [11-13]. Malignant cutaneous neoplasms arising in the hyperkeratotic skin of PLS patients are very rare [14,15]. To date, an association between Papillon-Lefevre syndrome and two cases of malignant melanoma has been reported [14,15]. No published report describing the development of nonmelanoma skin cancers within the hyperkeratotic lesions of the syndrome was found in the literature. We herein report a case of PLS, in which a squamous cell carcinoma (SCC) developed.

## Case presentation

**Figure 1.**
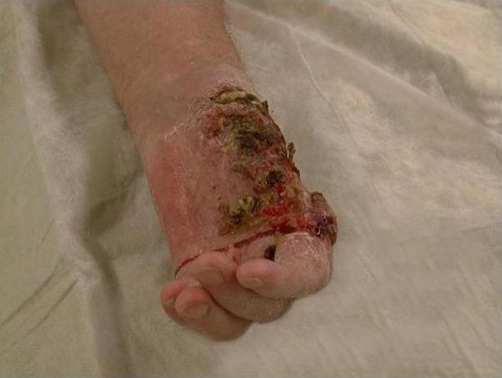
Two years after initial diagnosis, the non-treated SCC had significantly grown in size and was malodorous to the patient.

A 67-year-old German Caucasian male previously diagnosed with PLS was referred to our clinic complaining of a new lesion in his left palm. He was completely edentulous from the age of 15 years. Family history revealed a paternal cousin and one brother with PLS. Cutaneous lesions became markedly evident at the age of 1 year, in which the skin of the palms and soles was red, thickened, and painful and he now had severe palmoplantar hyperkeratosis. There was a history of hypercholesterolemia and hospitalisation at the age of 55 years and 63 years because of myocardial infarction. He had also been diagnosed as an alcohol abuser with liver cirrhosis and gastro-oesophageal reflux. A tissue biopsy confirmed the diagnosis of SCC in his left palm. He initially refused surgical treatment due to his perception of further loss of function and piano-playing potential in a hand, which already had a prior amputation of the ipsilateral thumb at the level of the metacarpophalangeal joint nine years previously due to osteomyelitis. Two years later, the SCC had significantly grown in size and was malodorous to the patient (Figure 1) and the hand was amputated. Two months later, there was a recurrence of the SCC and the distal half of the forearm was amputated. Unfortunately, within a further two months there was another local recurrence and he was diagnosed with two lung metastases. A palliative tumour debulking and median nerve neurolysis was performed but within a month there was a further local recurrence and the arm was disarticulated at the shoulder with simultaneous left axillary lymphadenectomy. The patient died two weeks later from his metastases.

## Discussion

**Figure 2.**
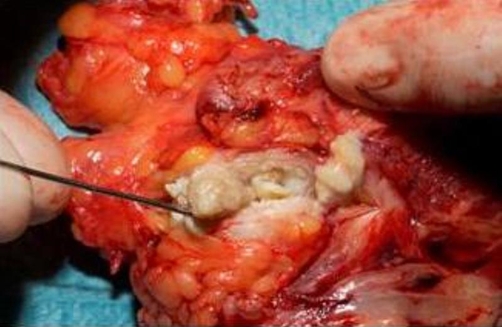
Macroscopic SCC invasion into a vein.

No published report describing the development of nonmelanoma skin cancer within the hyperkeratotic lesions of the syndrome has been reported in the literature, suggesting that this association is very rare. To date, only two cases of PLS associated with malignant cutaneous neoplasms, both malignant melanomas have been reported in the literature, in a Jew and a Japanese [14,15]. It has been reported that no association with malignant neoplasms, including malignant melanoma, was observed in 120 cases of PLS [16]. Various factors have shown significant association with SCC risks: evidence of sun damage, keratin horns, actinic keratoses, areas of leucoplakia and area of Bowen’s disease [17,18]. The pathogenic mechanism of SCC development during PLS remains to be elucidated. The authors postulate that chronic inflammation and altered keratinocytes in the hyperkeratotic lesions may play a role. An important aspect of this case was the 2 year history of a non-treated SCC due to the patient’s decision; the invasion into the veins and lymphatic vessels can be clearly seen in Figure 2. Two blinded specialists classified this tumor as pT3, N2, M2 and grade III. In contrast, other types of palmoplantar keratosis, such as Olmsted syndrome [19], and Huriez syndrome [20], have been reported to be associated with SCC. The literature contains recommendations for the treatment of the oral pathogies in PLS patients. In this case, the authors postulate that prompt excision of the SCC with adequate margins may have improved the outcome.

**Figure 3.**
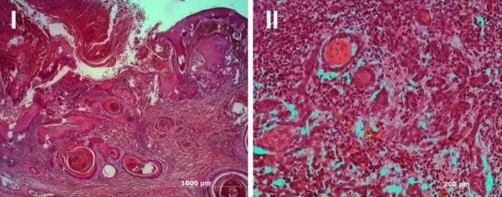
The non-treated SCC invading into lymphatic vessels **(I)** and veins **(II)**.

## Abbreviations

PLS: Papillon-Lefèvre syndrome
SCC: squamous cell carcinoma
